# Comparative assessment of antimicrobial, antiradical and cytotoxic activities of cannabidiol and its propyl analogue cannabidivarin

**DOI:** 10.1038/s41598-021-01975-z

**Published:** 2021-11-18

**Authors:** Chiara Russo, Margherita Lavorgna, Roberta Nugnes, Elena Orlo, Marina Isidori

**Affiliations:** Department of Environmental, Biological and Pharmaceutical Sciences and Technologies, University of Campania “Luigi Vanvitelli”, Via Vivaldi 43, 81100 Caserta, Italy

**Keywords:** Biological techniques, Cell biology

## Abstract

Cannabidiol and cannabidivarin are phytocannabinoids produced by *Cannabis indica* and *Cannabis sativa*. Cannabidiol has been studied more extensively than its propyl analogue cannabidivarin. Therefore, we performed a battery of in vitro biological assays to compare the cytotoxic, antiradical and antibacterial activities of both cannabinoids. Potential mitochondrial metabolism alterations, DNA synthesis inhibition, and plasma membrane damage were studied by MTT assay, BrdU-ELISA and LDH assay of cancer and normal human cells exposed to cannabinoids. ABTS and DPPH assays were performed to observe the effects of the cannabinoids on free radicals. Microbial susceptibility tests were performed to study the activity of the cannabinoids in two bacterial species implicated in human infections, *Escherichia coli* and *Staphylococcus aureus*. The results showed that the cannabinoids induced medium levels of cytotoxicity in cancer and normal cells at concentrations ranging from 15.80 to 48.63 and from 31.89 to 151.70 µM, respectively, after 72 h of exposure. Cannabinoids did not exhibit a strong antioxidant capacity in scavenging ABTS or DPPH radicals. No evident differences were observed between the two cannabinoids in antimicrobial activity, except with respect to *S. aureus*, which showed greater susceptibility to cannabidiol than to cannabidivarin after 72 h of exposure.

## Introduction

Cannabinoids and their propyl homologues are nonnitrogen-containing terpenophenols produced by *Cannabis indica* and *C. sativa*^1^. In addition to investigations on whole hemp plants and on the broad spectrum of molecules in phytocomplexes, a parallel increase in the number of scientific studies on the structure–activity relationship between the human endocannabinoid system and single cannabinoid molecules has increased during the past two decades (Fig. S1).

Cannabinoid molecules are able to bind cannabinoid receptors CB1 and CB2 belonging to the G protein-coupled receptor superfamily, and exert agonist/antagonist effects. CB1 is naturally expressed throughout the central nervous system, lungs, liver, and kidneys, while CB2 is found mainly in the immune system and in haematopoietic cells^2–4^. Both receptors are part of the endocannabinoid system involved in regulating physiological and cognitive processes. Except for the main psychoactive component of cannabis, Δ9-tetrahydrocannabinol (THC), other components, such as cannabidiol (CBD), cannabidivarin (CBDV), 9-tetrahydrocannabivarin (Δ9-THCV), cannabigerol (CBG), cannabichromene (CBC) and cannabivarin (CBV), lack psychotropic properties, and they have been studied for possible medical uses^5,6^. Among these phytocannabinoids, CBD and its propyl analogue, CBDV, are of interest since they are mainly involved in the modulation of receptors that are not in the endocannabinoid system. Research on CBDV has been limited, but CBD has been widely investigated as a cannabinoid with confirmed anticonvulsive, anti-inflammatory, analgesic, anti-nausea, anti-anxiety, antipsychotic, anti-epileptic, and antitumour properties, exerting no effect on normal cells^6–14^. In particular, the remarkable antioxidant properties of CBD have been associated with its neuroprotective activity^7,13^. Similarly, CBDV shows anticonvulsant and anti-epileptic properties without inducing psychoactive effects^15^, but no publications on its effect on redox balance or radical scavenging activity are available to date^16^. CBD has also been studied for its antimicrobial activity against gram-positive and gram-negative bacterial strains^17^ since the development of chronic inflammatory and degenerative diseases may be influenced by bacterial infections. In addition to numerous studies stating the beneficial pharmacological properties of CBD and some that identify those of CBDVs, publications have reported dangers of using CBD. Cannabidiol has been shown to induce teratogenicity^18^ and cytotoxicity in oligodendrocytes^19^. Furthermore, CBD suppresses the expression of skin differentiation genes, drives cells to acquire a less-differentiated state^20^ and acts via peroxisome proliferator-activated receptor gamma (PPARγ), which has been implicated in various pathological states, including adipogenesis, diabetes, atherogenesis, neurodegenerative disease, lung cancer and leukaemia^21–23^. Recently, Fisar and colleagues^24^ showed the CBD-induced inhibition of mitochondrial oxidative phosphorylation, involving calcium metabolism, while Ewing and colleagues^25^ demonstrated that CBD causes hepatotoxicity. In 2019, Tura and colleagues found that CBD induced pro-oxidant effects^26^. Another study showed that CBD epigenetically affected human development^21^, and another report indicated that it caused DNA damage and chromosomal aberrations in human hepatic- and buccal-derived cells^27^. Preparations containing CBD and CBDV are available in European countries and in the US (cannabis sales reached $15 billion globally in 2019; https://edition.cnn.com/2019/06/20/tech/cannabis-industry-15-billion/index.html) via the internet and in hemp shops, drugstores, and pharmacies^27^ with seemingly innocuous effects. In light of the contradictory scientific results, other studies on the biological effects of CBD and CBDV are required. Thus, the aim of this study was to increase data relating to CBD and CBDV through the performance of in-depth studies related to their effects on tumorous and normal cells, free radicals and bacterial growth. To identify the effects of CBD and CBDV, we investigated the potential alterations in mitochondrial metabolism, DNA synthesis inhibition, and plasma membrane damage using human-derived tumour cells and normal cells. The cancer cell lines chosen were alveolar basal epithelial adenocarcinoma cells (A549), epithelial colorectal adenocarcinoma cells (Caco-2), hepatoblastoma cells (Hep G2) and epithelial breast adenocarcinoma cells (MDA-MB-231). The normal cells were hTERT-immortalized skin fibroblasts (TelCOFS02MA). Potential alterations in the mitochondrial metabolic rate were detected by MTT assay. DNA synthesis inhibition was evaluated by BrdU-enzyme-linked immunosorbent assay (ELISA)^28^. Plasma membrane damage was assessed by lactate dehydrogenase (LDH) assay^29,30^. Furthermore, ABTS and DPPH assays were performed to observe the effects of CBD and CBDV on free radicals. Finally, a microbial susceptibility test was performed on two bacterial species involved in human infections: *Escherichia coli* (gram-negative) and *Staphylococcus aureus* (gram-positive). The data collected in this study will provide important information for both industry and regulatory agencies in regard to the short-term in vitro toxicity of CBD and CBDV. The results will aid in selecting appropriate models and concentrations for long-term in vivo studies.

## Results and discussion

### Cytotoxic effects

**Figure 1:**
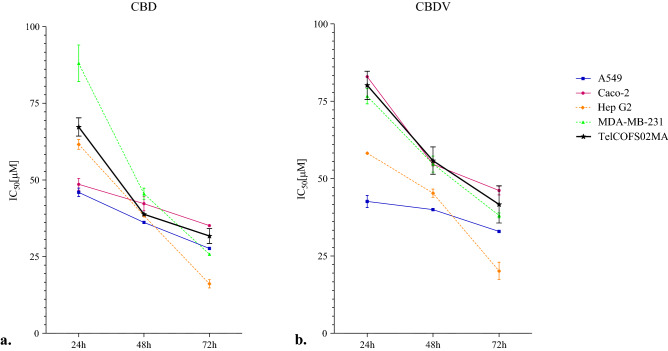
IC50 (µM) values obtained by MTT assay. IC50 (µM) values obtained by MTT assay of A549, Caco-2, Hep G-2, MDA-MB-231 and TelCOFS02MA cells after 24, 48, and 72 h of exposure: (**a**) CBD and (**b**) CBDV. The trends are based on the interpolation of five independent experiments using GraphPad Prism 5 software. The bars represent the standard deviation of the mean value obtained from five experiments.

The results from MTT assays were obtained after 24, 48, and 72 h of exposure to CBD and CBDV and are reported in Fig. 1 and Table S1. For all cell lines, the lowest IC50 values were recorded after 72 h of treatment.

Specifically, after 72 h of treatment, the highest sensitivity to CBD and CBDV was observed in HepG2 cells, with IC50 values equal to 15.80 and 19.74 µM, respectively (Fig. 1 and Table 1); however, higher median concentrations for inhibiting mitochondrial metabolism were observed after only 24 h and 48 h of treatment (Fig. 1).

**Table 1:** IC50 values obtained by MTT assay, BrdU-ELISA and LDH assay.

Cell line	MTT		BrdU-ELISA		LDH	
	CBD	CBDV	CBD	CBDV	CBD	CBDV
A549	27.66 (23.82–32.12)	32.91 (27.89–38.83)	40.38 (29.08–56.05)	48.63 (33.94–69.67)	23.96 (18.47–31.09)	26.30 (24.11–28.69)
Caco-2	35.24 (28.23–43.98)	46.02 (40.01–52.93)	33.32 (25.41–43.69)	40.39 (29.78–54.85)	22.75 (16.12–32.12)	29.09 (23.55–35.94)
Hep G2	15.80 (11.33–22.04)	19.74 (13.86–28.10)	39.21 (31.60–48.64)	46.68 (39.81–54.75)	18.14 (14.72–22.36)	21.16 (17.49–25.60)
MDA-MB-231	25.84 (23.61–28.28)	38.09 (32.39–44.83)	38.38 (34.50–42.68)	46.82 (37.29–58.83)	22.13 (18.25–26. 82)	33.77 (26.37–43.25)
TelCOFS02MA	31.89 (25.05–40.61)	42.30 (26.93–66.44)	49.14 (32.71–73.83)	69.14 (55.39–86.31)	118.00 (39.10–356.10)	151.70 (58.10–396.20)

MTT assay, BrdU-ELISA and LDH assay IC50 values, expressed in µM, with confidence intervals of 95% for the cell lines after 72 h of exposure to CBD and CBDV.

**Figure 2:**
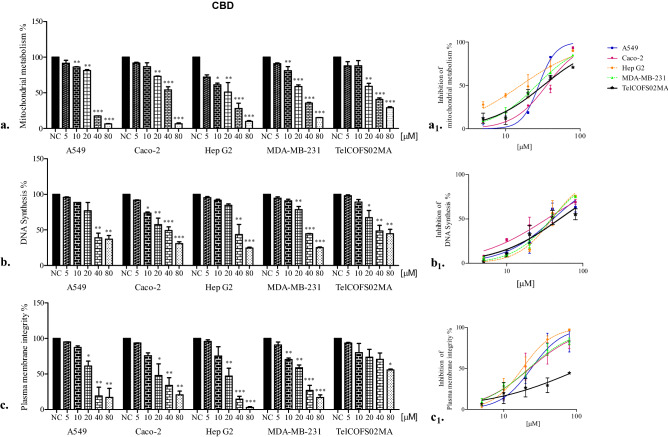
Percentages of cellular mitochondrial metabolism, DNA synthesis and plasma membrane integrity after 72 h of CBD exposure. Percentages of cellular mitochondrial metabolism (**a**), DNA synthesis (**b**) and plasma membrane integrity (**c**) after 72 h of exposure of the A549, Caco-2, HepG2, MDA-MB-231, and TelCOFS02MA cell lines to different concentrations of CBD. The results are expressed as the means ± standard deviation on the basis of five independent experiments. Significant differences compared to negative controls are highlighted by asterisks (ANOVA, Dunnett’s test—*p < 0.05; **p < 0.001; ***p < 0.0001)*.* Percentages of inhibition of cellular mitochondrial metabolism (**a**_1_), DNA synthesis (**b**_1_), and inhibition of plasma membrane integrity (**c**_1_) after 72 h of exposure are reported in concentration [µM]-effect curves. NC: negative control.

**Figure 3:**
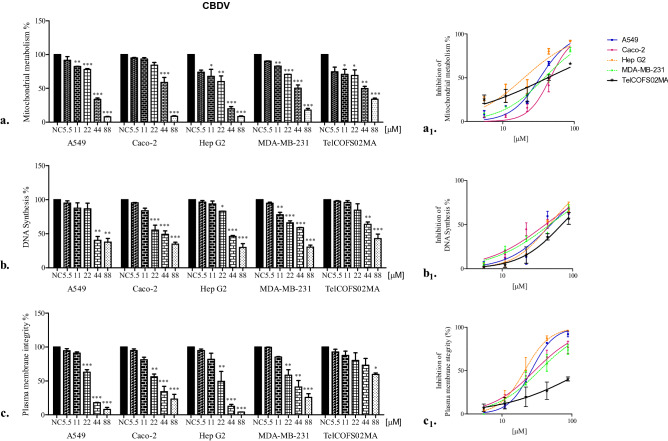
Percentages of cellular mitochondrial metabolism, DNA synthesis and plasma membrane integrity after 72 h of CBDV exposure. Percentages of cellular mitochondrial metabolism (**a**), DNA synthesis (**b**) and plasma membrane integrity (**c**) after 72 h of exposure of the A549, Caco-2, HepG2, MDA-MB-231, and TelCOFS02MA cell lines to different concentrations of CBDV. The results are expressed as the means ± standard deviations on the basis of five independent experiments. Significant differences compared to the negative controls are highlighted by asterisks (ANOVA, Dunnett’s test—*p < 0.05; **p < 0.001; ***p < 0.0001). Percentages of inhibition of cellular mitochondrial metabolism (**a**_1_), inhibited DNA synthesis (**b**_1_), and inhibition of plasma membrane integrity (**c**_1_) after 72 h of exposure are reported in concentration [µM]-effect curves. NC: negative control.

In contrast, after 72 h of exposure, the least affected mitochondrial activity was observed in Caco-2 and TelCOFS02MA cells, as shown in Table 1 (35.24 and 46.02 µM IC50 for CBD and CBDV in Caco-2 cells, respectively, and 31.89 and 42.30 µM IC50 for CBD and CBDV in on TelCOFS02MA cells, respectively). As shown in Figs. 2 and 3, effective percentages were reported in addition to the significantly lowest adverse effect concentrations (LOAECs), which were obtained by statistical analysis after 24, 48 and 72 h of treatment (LOAECs obtained after 72 h of treatment are reported in Figs. 2 and 3).

For Caco-2 cells, the same LOAEC value (20 µM, CBD; 44 µM, CBDV) was observed after each exposure time, while for TelCOFS02MA cells, the same LOAEC (20 µM, for the entire treatment period) was observed only after CBD exposure, while CBDV treatment led to a concentration–time-dependent trend: 88 µM (24 h), 44 µM (48 h) and 11 µM (72 h). After 72 h of treatment, both cannabinoids affected the mitochondrial metabolism of cells similarly, except for MDA-MB-231 cells. As shown in Table 1, CBD had a greater impact on breast cancer cells, with IC50 values, as well as confidence intervals, lower than those observed for CBDV. This difference was also observed for LOAEC values obtained after 24 and 48 h of exposure, which were higher for CBDV (44 and 22 µM, respectively) than for CBD (20 and 10 µM, respectively). No significant difference was observed between the negative control and solvent control.

The data obtained in this study may be compared with those reported in the literature. Choi and collaborators^31^ studied the in vitro cytotoxic effects of CBD based on reduced cellular metabolism by using MTT in NIH3T3 fibroblasts, A549 lung cancer cells, MDA-MB-231 breast cancer cells, and SNU-C4 colon cancer cells and obtained IC50 values ranging from 29.04 to 36.70 µM after 48 h of exposure, which is at the same order of magnitude as the results obtained in this study (Fig. 1). Choi et al.^31^ explained that CBD-induced cytotoxicity increased in a dose- and time-dependent manner, causing cell morphological changes, formation of cell protuberance and changes in membrane blebs. In addition, as reported by Fišar and colleagues^24^, CBD was able to inhibit the mitochondrial respiration rate, linking the effect to CB1 receptors localized on mitochondrial membranes. Moreover, Kalenderoglou and colleagues^32^ found that CBD induced a reduction in cell viability, both in terms of reduction in mitochondrial respiration and in the overall number of lymphoblastic Jurkat T cells when tested at concentrations ranging from 1 to 100 µM. In addition, in 2019, Olivas-Aguirre et al.^33^ noticed that CBD directly targeted mitochondria, altering calcium homeostasis and causing mitochondrial Ca^2+^ overload in acute lymphoblastic leukaemia and provoking stable mitochondrial transition pore formation, ROS production, autophagy and cell death.

To the best of our knowledge, only one study has been performed to test the effect on CBDV on human cells by MTT: in 2016, Olah and colleagues^34^ used SZ95, normal-like sebocytes, obtaining a significant decrease in cell viability at 100 nM after 24 h of treatment, which is 2–3 orders of magnitude lower than those found in this study.

Regarding DNA synthesis inhibition, median concentrations were obtained by BrdU-ELISA after 72 h of treatment (Table 1). The lowest IC50 values were observed for Caco-2 cells, which were 33.32 and 40.39 µM for CBD and CBDV, respectively, while the highest median concentrations were observed for TelCOFS02MA cells (49.14 and 69.14 µM, respectively, for CBD and CBDV). Essentially, after CBD treatment, no differences in 95% confidence intervals were noted among cell lines, in contrast to CBDV treatment, after which the 95% confidence intervals of the Caco-2 and Hep G2 cell lines did not overlap. The lower sensitivity of fibroblasts to CBDV was supported by LOAEC values (22 µM for Caco-2 and Hep G2 cells vs. 44 µM for TelCOFS02MA cells) (Fig. 2). In fact, as early as 1980, Samson and Schwartz^35^ collected evidence of an adaptive DNA repair pathway in human skin fibroblast lines after DNA damage.

Furthermore, 72 h of CBD treatment affected DNA synthesis, showing median cytotoxic effects at concentrations with confidence intervals overlapping with those obtained by MTT assay. CBD affected mitochondrial metabolism in HepG2 and MDA-MB-231 cells to a greater extent than it affected DNA synthesis, and CBDV affected mitochondrial metabolism in HepG2 cells (Table 1, Figs. 2 and 3).

McAllister and collaborators^36^ remarked that cannabidiol is able to inhibit Id-1 (an inhibitor of differentiation/DNA binding) gene expression in aggressive breast cancer cells, leading to the attenuation of tumour aggressiveness. Furthermore, Jeong et al.^8^ found that CBD induced the inhibition of DNA synthesis and apoptosis of human colorectal cancer cells (CRC) by increasing the expression of Bcl-2 (B-cell lymphoma 2) homology 3 domain-only protein (Noxa). Moreover, as reported by Zhang et al.^14^, CBD significantly upregulates ataxia telangiectasia-mutated (ATM) gene and p53 protein expression and downregulates p21 protein expression in cancer cells, reducing the levels of CDK2 and cyclin E with cell cycle arrest in the G0–G1 phase. However, no evidence for the inhibition of DNA synthesis by CBDV in human cells has been reported in the literature.

Cytotoxicity related to membrane integrity loss has been previously investigated via LDH assay^29,30^. Moreover, all cell lines were similarly affected by CBD and CBDV after 72 h of exposure, and the lowest IC50 values were observed for HepG2 cells (18.14 and 21.16 µM, respectively, for CBD and CBDV, Table 1). Only TelCOFS02MA cells were insensitive to cannabinoids with respect to membrane damage, with IC50 values equal to 118 and 151.7 µM and LOAEC values equal to 80 and 88 µM for CBD and CBDV, respectively. Reddy and collaborators^37^ demonstrated that lysosomal Ca^2+^-regulated exocytosis in wounded mouse skin fibroblasts was critical for plasma membrane repair. In 2008, Choi and colleagues^31^ noticed that immortalized mouse embryonic fibroblasts as well as human lung, breast and colon cancer cells exposed to 5–80 μM CBD for 24 h exhibited an increase in LDH release and that the cytotoxicity of CBD increased in a dose-dependent manner. Cerretani et al.^38^ observed a CBD-cytotoxic effect in HT-29 colorectal adenocarcinoma cells after 24 h of treatment, as indicated by LDH assay, leading to the conclusion that cytotoxicity was induced through a CB1 and CB2 receptor-independent mechanism (with experiments using the CB1 antagonist AM251 and CB2 antagonist AM630) and, possibly, via ROS production leading to apoptotic cell death. In addition, when CBD (1–20 µM) was tested on a myeloma cell line for 24 h, the level of LDH was significantly increased^39^. CBD may confer protection against cytokine-induced killer (CIK) cells, which constitute a heterogeneous T cell population capable of exerting potent major histocompatibility complex (MHC)-unrestricted cytotoxicity against both haematologic and solid tumours^39^.

**Figure 4:**
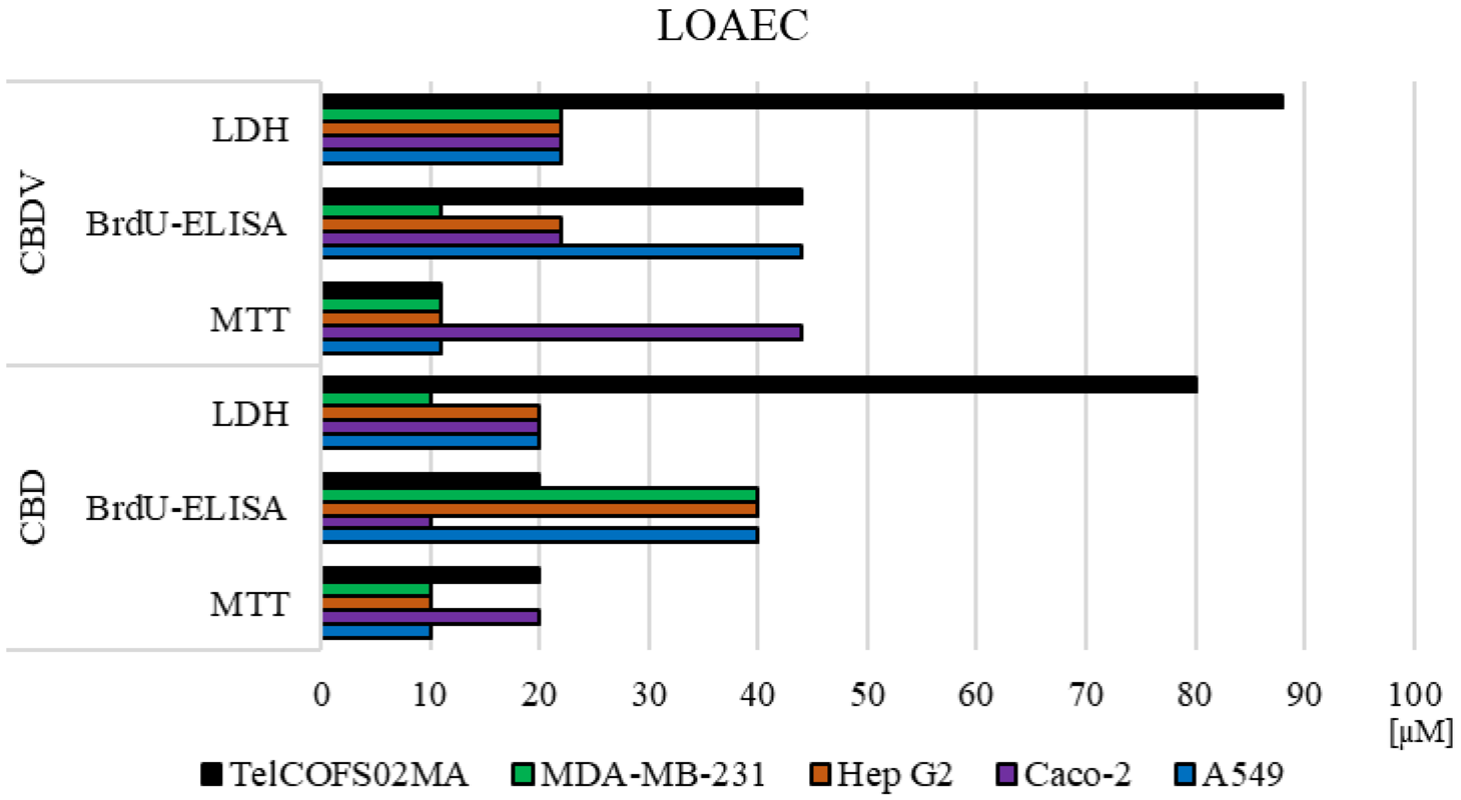
LOAECs values. LOAEC values after 72 h of exposure (ANOVA, Dunnett’s test—*p < 0.05).

To compare the results obtained in the three cytotoxicity tests, the lowest significant adverse effect concentrations after 72 h of CBD and CBDV exposure were statistically calculated for each assay and for each cell line. The results are depicted in Fig. 4.

It is evident that in A549 cells, CBD and CBDV affected mitochondrial metabolism at lower concentrations than those required to affect membrane integrity and DNA synthesis. In contrast, mitochondrial metabolism and DNA synthesis in Caco-2 cells were affected by CBD at lower concentrations than CBDV. However, CBDV was shown to be more effective than CBD on DNA synthesis in HepG2 cells. The opposite trend was observed in MDA-MB-231 and TelCOFS02MA cells. In the MDA-MB-231 cells, CBD exerted a greater effect on membrane integrity, while CBDV exerted a greater effective on DNA synthesis; in TelCOFS02MA cells, CBD exerted a greater effect on DNA synthesis, and CBDV exerted a greater effect on mitochondrial metabolism.

There is no doubt that different transduction pathways may play a key role in the differences in cytotoxicity observed in different cell lines. In fact, Pagano et al.^40^ explained that CBD displays inverse agonism to the human CB2 receptor and may interact with transient receptor potential vanilloids (TRPVs), serotonin 1A G-protein-coupled receptor (5-HT1A), G-protein-coupled receptor 55 (GPR55), and PPARγ, inducing apoptosis and inhibiting cell migration and metastasis in different cancer types.

Specifically, Kim et al.^41^ demonstrated that cannabidiol enhanced tumour necrosis factor-related apoptosis-including ligand (TRAIL) expression, inducing the apoptosis of colorectal cancer cells.

Milian et al.^42^ observed that, in lung cancer cells, CBD inhibited the expression of epidermal growth factor receptor (EGFR), cell proliferation, and the epithelial-to-mesenchymal transition (EMT), thereby affecting the cellular cytoskeleton and reducing in vitro migration.

According to Ligresti et al.^11^ and Fonseca et al.^43^, cannabidiol induced the apoptosis of human breast cancer cells via activation of cannabinoid CB2 and TRPV1 receptors and increasing intracellular calcium and ROS/RNS generation. Additionally, Shrivastava et al.^44^ observed that in human breast cancer cells, CBD caused ER stress and, subsequently, the inhibition of AKT and mTOR signalling, as shown by decreased levels of phosphorylated mTOR, 4E-binding protein 1, and cyclin D1, highlighting the intricate interplay between apoptosis and autophagy in CBD-induced breast cancer cells. According to Ford et al.^45^, MDA-MB-231 breast cancer cells are involved in highly active cellular signalling pathways associated with GPR55 and L-a-lysophosphatidylinositol (LPI), suggesting potential alterations in the cytoskeleton, cell morphology and cell migration after CBD exposure.

According to Fonseca^43^, a foreskin fibroblast line expresses all elements of the endocannabinoid system, as well as the receptor TRPV1; nevertheless, no LDH release was observed.

In addition, according to Lim^46^, CBD provokes an endoplasmic reticulum (ER) stress response, with changes in its morphology and the initiation of RNA-dependent protein kinase-like ER kinase activity, and induces downstream activation of the pro-apoptotic IRE1/ASK1/c-Jun N-terminal kinase pathway, leading to hepatic HSC cell death. In 2003, Li et al.^47^ reported that PPARγ ligands (such as CBD) can induce apoptosis and inhibit the proliferation of HepG2 cells.

### Activity in the presence of free-radicals

The concentrations of CBD, CBDV and Trolox able to reduce the initial absorbance of ABTS or DPPH radicals by 50% are reported in Table 2. CBD and CBDV showed similar EC50 values, on the order of hundreds of μmol/L and thousands of μmol/L, respectively, for scavenging ABTS and DPPH radicals. No statistically significant differences were found when comparing the solvent control to the negative control.

**Table 2:** EC50 values.

	ABTS	DPPH
TROLOX	111.2 (96.8–127.6)	326.9 (277.2–385.4)
CBD	528.4*** (382.1–730.9)	3055.0*** (1697.0–5499.0)
CBDV	406.3** (344.9–478.7)	3736*** (3284.0–4250.0)

EC50 values with confidence limits (95% probability, in brackets), expressed in µM, obtained by ABTS and DPPH assays testing Trolox, CBD and CBDV effectiveness. Dunnett's multiple comparison test was performed with the cannabinoids and Trolox results: **p < 0.001 and ***p < 0.0001.

**Figure 5:**
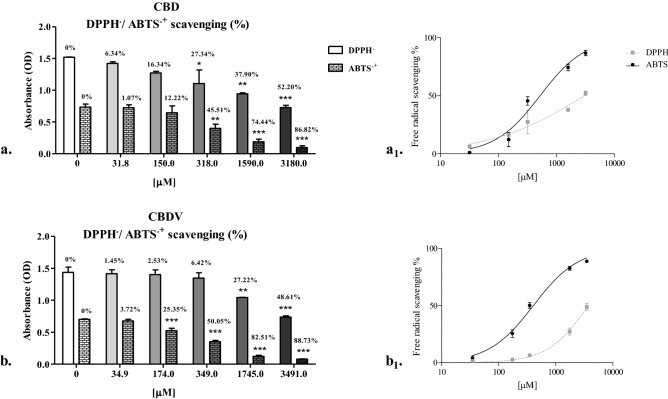
DPPH /ABTS+ scavenging (%). DPPH/ABTS+ scavenging (%) at different concentrations (µM) of CBD (**a**) and CBDV (**b**) as determined by DPPH and ABTS assays. The results are expressed as the means ± standard deviations obtained from five independent experiments. Dunnett's multiple comparison test was performed with OD values of the negative control (0 µM) and all other tested concentrations: *p < 0.05, **p < 0.001, ***p < 0.0001. The percentage of free radicals scavenged by CBD (**a**_1_) and CBDV (**b**_1_) is reported in concentration [µM]-effect curves.

Our results are partially consistent with findings observed by Kitamura et al.^48^, who performed ABTS and DPPH assays on CBD oil samples and obtained EC50 values ranging from 67 to 138 µM in the ABTS assay and from 522 to 1075 µM in the DPPH assay, concentrations reaching the same order of magnitude as that reported for the concentrations obtained in the present study. Interestingly, in line with the results of Floegel and colleagues^49^, the antioxidant capacity detected by ABTS assay was significantly higher for phytoderivatives than that detected by DPPH assay, as reported in Fig. 5.

Comparing the effects of CBD and CBDV to the effect of Trolox, a significant reduction (p < 0.001, p < 0.0001) in the scavenging activity for both radicals was observed. In comparison with the Trolox equivalent antioxidant capacity (TEAC) value, CBD and CBDV showed ABTS scavenging capacities that were 79 and 73% less, respectively, than the capacity of Trolox (TEAC CBD = 0.21 and TEAC CBDV = 0.27), and the DPPH scavenging capacities of CBD and CBDV were 90 and 91% less, respectively, than the capacity of Trolox (TEAC CBD = 0. 1, and TEAC CBDV = 0.09). Notably CBD is able to reduce free radical generation and lipid peroxidation in a cellular environment due to its ability to increase the mRNA expression of copper/zinc superoxide dismutase, a key endogenous enzyme that reduces oxidative stress, contributing to cell survival, as explained by Garcia-Arencibia et al.^50^ and Moldzio and colleagues^51^. Nevertheless, in this study, little free radical scavenging activity was observed in vitro without a cellular substrate. On the other hand, pro-oxidant activity can often be greater than antioxidant activity, which may explain the cytotoxicity shown here by cannabinoids in human cells, as determined by MTT assay, BrdU-ELISA and LDH assay. Hence, the stress induced by CBD may be linked to alterations in mitochondrial homeostasis leading to caspase activation involved in the intrinsic and extrinsic pathways of ROS-induced apoptosis^38,43^. Thus, the production of ROS is the most supported hypothesis for the CBD-dependent inhibition of cancer cell aggressiveness in experimental models of human cell cultures^38^.

### Activity in the presence of bacteria

The results did not show evident differences in *E. coli* susceptibility to the two selected cannabinoids (Figs. S2 and S3), with IC50 values equal to 29.10 (24.58–34.44) and 35.47 (27.20–46.30) µM for CBD and CBDV, respectively, after 72 h of incubation. On the other hand, *S. aureus* was more susceptible to CBD than CBDV. Specifically, the CBD IC50 values were equal to 10.27 (9.48–11.10), 4.86 (4.45–5.28) and 1.84 (1.56–2.16) µM after 24, 48 and 72 h of exposure, respectively. The IC50 value obtained after exposing *S. aureus* to CBDV for 72 h was equal to 30.83 (23.92–39.73) µM, one order of magnitude higher than the IC50 value obtained after CBD exposure for 72 h. No statistically significant differences were found when comparing the solvent control to the negative control. These findings were in line with those of Karas and colleagues^17^, who reported that cannabinoids were more active against G^+^ than G^−^ strains. In 1976, Van Klingeren and collaborators^52^ tested CBD against *S. aureus,* finding the minimum inhibiting concentration (MIC) within a range between 3.18 and 15.90 µM after overnight exposure. Later, in 2020, Martinenghi and coauthors^53^ tested CBD against several G^+^ pathogens, observing MIC values ranging from 3.18 to 6.36 µM. In addition, in 2020, Pellegrini and coauthors^54^ tested the effect of hemp essential oil on *S. aureus* and found MIC values ranging from 3.97 to 15.90 µM. According to Wassmann and coauthors^55^, to date, little is known of the antimicrobial mechanism of action of cannabinoids; nevertheless, these authors explained that exposing *S. aureus* to CBD decreased the membrane potential. In addition, Blaskovich and coauthors^56^ found that protein, DNA, RNA and peptidoglycan synthesis were inhibited when *S. aureus* was exposed to CBD at 6–14 µM, causing a rapid bactericidal action, shutting down all these synthesis pathways and resulting in more activity towards gram-positive bacteria than gram-negative bacteria.

As suggested by El-Mosalamy and colleagues^57^, urinary bladder infection by *E. coli* may play a major additive and synergistic role during bladder carcinogenesis. In addition, De la Calle and co-authors^58^ reported that *S. aureus* infection is the most frequent comorbidity with chronic lung disease (34.7%), chronic renal failure (31.6%), diabetes mellitus (29.6%), and cardiovascular disease (31.6%). To the best of our knowledge, the antimicrobial activity of CBDV is still not well understood.

## Materials and methods

### Reagents

5-Bromo-1-(2-deoxy-β-d-ribofuranosyl) uracil (5-BrdU, CAS: 59-14-3), a fixing/denaturing solution (FixDenat, Cat# 11758764001), anti-BrdU- peroxidase (POD) from mouse IgG1_clone BMG6H8 (Cat# 11585860001), 2-methyl-4-isothiazolin-3-one hydrochloride (CAS: 26172-54-3), lead(II) sulfide (PBS, CAS: 1314-87-0), 3,3′,5,5′-tetramethylbenzidine (TMB, CAS: 54827-17-7), diaphorase (CAS: 9001-18-7); Triton X-100 (CAS: 9002-93-1), and 2,3,5-triphenyltetrazolium chloride (CAS: 146-68-9) were purchased from Roche (Sigma-Aldrich), Milan, Italy. 6-Hydroxy-2,5,7,8-tetramethylchromane 2-carboxylic acid (Trolox, CAS: 53188-07-1), 2,2′-diphenyl-1-picrylhydrazyl (DPPH, CAS: 1898-66-4), 2,2′-azino-bis-(3-etilbenzotiazolino-6-sulfonic acid) (ABTS, CAS: 30931-67-0), potassium persulfate (CAS: 7727-21-1), 3-(4,5-dimethylthiazol-2-yl)-2,5-diphenyltetrazolium bromide (MTT, CAS: 298-93-1), and 2-propanol were purchased from Sigma-Aldrich (Milan, Italy). Roswell Park Memorial Institute medium (RPMI) 1640, Dulbecco’s modified Eagle’s medium (DMEM, with phenol red and phenol red-free), Dulbecco’s phosphate-buffered saline (DPBS), l-glutamine, trypsin–EDTA, N-(2-hydroxyethyl)piperazine-N′-(2-ethanesulfonic acid) (HEPES), penicillin/streptomycin (10,000 U/mL), foetal bovine serum (FBS), and nonessential amino acids (MEM, 100×) were purchased from Lonza Bio Whittaker (Verviers, Belgium).

### Test compounds

Cannabidiol (CBD, CAS: 13956-29-1, purity 99.95%) was obtained from LGC Standards GmbH (Germany), and cannabidivarin (CBDV, CAS: 24274-48-4, purity of 99.80%) was obtained from Sigma-Aldrich (Milan, Italy). Both compounds were dissolved in methanol.

### Measurement of cytotoxic effects

#### Cultivation of human cell lines

The alveolar basal epithelial adenocarcinoma (A549) and colon adenocarcinoma (Caco-2) cell lines were provided by Prof. Pacifico and Prof. Potenza (Department of Environmental, Biological and Pharmaceutical Sciences and Technologies, University of Campania “Luigi Vanvitelli”, Italy), respectively; the hepatoblastoma cell line (Hep G2) and epithelial breast adenocarcinoma cell line (MDA-MB-231) were provided by Prof. Abbondanza (Department of Precision Medicine, University of Campania “Luigi Vanvitelli”, Italy), respectively. The hTERT-immortalized dermal fibroblast cell line (TelCOFS02MA, CRL­4005™) was purchased from ATCC (Milan, Italy).

A549, Caco-2, HepG2, and MDA-MB-231 cells were maintained in Roswell Park Memorial Institute Medium (RPMI 1640). TelCOFS02MA cells were maintained in Dulbecco’s modified Eagle’s medium (DMEM) with 1.0 mM sodium pyruvate. All cell growth media were supplemented with 10% FBS, 2% l-glutamine, 2% HEPES and 1% penicillin/streptomycin (10,000 U/mL). For Caco-2 cell cultures, 1% MEM 100× was added to the growth medium. All cells were stored in liquid nitrogen. Passages (from the fifth to the tenth) were used for cytotoxicity experiments. The establishment of the biochemical and morphological identity and purity of the cell lines is one of the contributing factors to the reproducibility of research results. Periodic quality control testing procedures (authentication, characterization and mycoplasma testing) were performed. Cells were cultured in T-75 tissue culture flasks (Sarstedt, Verona, Italy) in a humidified atmosphere of 95% air plus 5% CO_2_ at 37 °C. The media were changed every 2–3 days. After reaching 80–90% confluence, the cells were washed with DPBS, detached with trypsin–EDTA, centrifuged (200*g*, 5 min, 21 °C), counted with the vital dye trypan blue using an optical microscope and subcultured.

#### MTT assay

MTT assay is the most commonly used method for high-throughput identification of the antiproliferative effects of compounds on cultured cells and is indirectly correlated with mitochondria-dependent apoptosis progression^59–61^. The production of reducing equivalents such as NADH in metabolically active cells is an indicator of “cell redox activity”, indirectly reflecting the number of viable cells^60,61^ and the mitochondrial metabolic rate in cells^62,63^.

A549, Caco-2, HepG2 and TelCOFS02MA cells (1 × 10^4^ cells/well), and MDA-MB-231 cells (0.5 × 10^4^ cells/well) were seeded in 100 µL of fresh medium into 96-well plates (TC-Plate 96 wells, Sarstedt, Verona, Italy) in quadruplicate for 24 h at 37 °C in a humidified atmosphere of 95% air plus 5% CO_2_. Then, the medium was removed and replaced with 200 µL of media with different concentrations of CBD (5–80 µM, dilution factor [DF] equal to 2) and CBDV (5.5–88 µM, DF = 2) chosen on the basis of range-finding tests (0.5, 5, and 50 mg/L). We chose 1.56–25 mg/L to be tested and, to make easier the comparison between the two cannabinoids activities, the concentrations were reported in [μM]. In all experiments, negative controls (wells containing cells exposed only to the medium) and solvent controls (wells containing cells exposed to 2.5% methanol) were included. The plates were incubated at 37 °C for three exposure times: 24, 48 and 72 h (different plates were prepared for each specific time point). Then, MTT solution (5 mg/mL) was added to each well, and the plates were re-incubated for 4 h at 37 °C. The culture medium was removed, and 2-propanol was used to dissolve formazan crystals. The absorbance of formazan was measured spectrophotometrically at 590 nm using an automated microplate reader (Synergy H1, Biotek, Winooski, USA). The results are reported as the percentages of cells with inhibited proliferation, as determined by the link between the inhibition of mitochondrial metabolic activity and proliferation. The results were calculated with the following formula:

1 $$IC\left(\%\right)=1-\left[\frac{{OD}_{590} \; sample}{{OD}_{590} \; negative \; control}\right] \cdot 100$$

Furthermore, IC50 values (concentrations of the sample used to obtain an inhibition of 50% of cell growth/metabolic activity) were calculated.

#### BrdU-ELISA

BrdU-ELISA was performed according to the protocol described by Lehmann and collaborators^64^, and the results were used to evaluate the inhibition of DNA synthesis^65^. A total of 1 × 10^4^ cells/well were seeded in 100 µL of fresh medium into 96-well plates in triplicate at 37 °C in a humidified atmosphere of 95% air plus 5% CO_2_ for 24 h. Then, the medium was removed and replaced with 200 µL of medium with the same concentrations of CBD and CBDV that had been used in the previous test. In all experiments, blanks (wells with medium and without cells), negative controls (with only medium) and solvent controls (2.5% methanol) were included. The plates were incubated at 37 °C for 72 h. Then, 20 µL of 100 µM BrdU labelling solution was added to each well and incubated for 2 h at 37 °C. After removing the labelling solution, 200 µl of DNA fixing/denaturing solution was added to each well and incubated for 30 min at room temperature. After its discharge, 100 µL of the anti-BrdU-POD antibody solution [0.075 U/mL] was added to each well and incubated for 90 min at room temperature. After several washing steps with 1X PBS (200–300 µL/well), 100 µl of tetramethylbenzidine (TMB) was added to each well and incubated for 20–30 min (sufficient time for the colour change). The incorporation of BrdU into DNA was quantified spectrophotometrically at 370 nm. Blank wells revealed information about the nonspecific binding of BrdU and the anti-BrdU-POD conjugate to microplates, and their absorbance (less than 0.1) was subtracted from all other values. The mean absorbance of the control cells represented 100% cell proliferation, and the mean absorbance of the treated cells was related to the control values to determine cell sensitivity. Inhibition (%) of DNA synthesis/cell viability was determined as follows:

2 $$\text{IC}\left({\%}\right)=1-\left[\frac{{\text{OD}}_{370} \; \text{ sample}}{{\text{OD}}_{370} \; \text{ negative} \; \text{control}}\right] \cdot 100$$

IC50 values (representing the median concentration at which DNA synthesis and cell viability were reduced by 50%) were calculated.

#### LDH assay

LDH assay is a high-throughput method based on the release of the LDH cytoplasmic enzyme from the cytosol into the extracellular space when the plasma membrane is damaged^29,30^. Plasma membrane damage related to the amount of lysed cells was monitored by LDH assay, and the results were used for indirectly detecting the necrosis correlated to the leakage of the cytosolic enzyme due the loss of membrane permeability control^29,30^. This assay was performed with the protocol described by Lehmann and colleagues^64^.

Briefly, 1 × 10^4^ A549 and Caco-2 cells and 2 × 10^4^ HepG2, MDA-MB-231 and TelCOFS02MA cells were seeded in 100 µL of 1% FBS-fresh medium in each well of 96-well plates and incubated for 24 h at 37 °C with 95% humidity and 5% CO_2_. Then, the medium was removed and replaced with 100 µL of medium with different concentrations of CBD and CBDV, which were chosen on the basis of range-finding tests (as previously indicated). The plates were incubated at 37 °C for 72 h. In all experiments, the background control (wells with medium and without cells to obtain information about the LDH activity in the assay medium), low control (wells containing cells exposed only to the medium to determine spontaneous LDH release), high control (wells containing cells exposed to 2% Triton-X100 for 10 min before performing the assay to determine maximum LDH release), and solvent controls (2.5% methanol) were included. One hundred microlitres of supernatant was removed carefully from each well and transferred into corresponding wells in optically clear 96-well flat bottom microplates. One hundred microlitres of freshly prepared reaction mixture (Diaphorase (Catalyst)/NAD^+^ /INT/sodium lactate) was added to each well and incubated for 30 min at 20 °C in darkness. Thus, NAD^+^ was reduced to NADH/H^+^ by the LDH-catalysed conversion of lactate to pyruvate. Then, the catalyst transferred H/H^+^ from NADH/H^+^ to the tetrazolium salt INT, which was reduced to formazan. To arrest the reaction, 50 µL/well of 1 N HCl was added. The spectrophotometric absorbance was determined at 492 nm. The absorbance of the background control wells was subtracted from all other values.

The results are reported as percentages of cytotoxicity, which is linked to the percentage of LDH released from damaged plasma membranes, calculated as follows:

3 $$\text{IC}\left({\%}\right)=\frac{{\text{OD}}_{492} \; \text{ sample}-{\text{OD}}_{492} \; \text{ low} \; \text{control}}{{\text{OD}}_{492}\text{ high} \; \text{control}-{\text{OD}}_{492} \; \text{ low} \; \text{control}}\cdot 100$$

IC50 values (concentrations of the sample to obtain 50% cytotoxicity) were calculated.

### Activity in the presence of free-radicals

#### ABTS assay

An ABTS assay was performed following the procedure described by Lavorgna et al. 2019^66^. The cationic ABTS radical (ABTS^+^) was generated by oxidizing ABTS (7 mM) with potassium persulfate (140 mM K_2_S_2_O_8_), and the mixture was incubated for 12–16 h in the dark. The ABTS^+^ working solution was diluted to an absorbance of 0.7 ± 0.2 OD at 734 nm. Thus, the negative control was generated with 1 mL of ABTS^+^ solution and 100 µL of distilled water, and the samples were prepared with 1 mL of ABTS^+^ solution and 100 µL of different concentrations of CBD (31.8, 150, 318, 1590, or 3180 µM) and CBDV (34.91, 174, 349, 1745, or 3491 µM), which were chosen on the basis of range finding tests. Distilled water was used for the blank, Trolox was used for the standard control, and methanol was used for the solvent control. The absorbance was measured at 734 nm after 6 min, and the percent decrease in free radical absorbance was calculated as suggested by Rakholiya et al.^67^:

4 $$\text{Inhibition }\left({\%}\right)=\frac{{\text{OD}}_{734}\text{radical}- {\text{OD}}_{734}\text{sample }}{{\text{OD}}_{734}\text{radical}} \cdot 100$$

The results are expressed as the median effective concentration (EC50). Furthermore, Trolox equivalent antioxidant capacity (TEAC) values were calculated following the procedure used by Shimamura end colleagues^68^:

5 $$\text{TEAC }=\frac{\text{EC}50 \; \text{ Trolox }}{\text{EC}50 \; \text{ sample}}$$

#### DPPH assay

DPPH assay was performed following the protocol described by Brand-Williams et al.^69^. DPPH^⋅^ was dissolved in methanol, generating a purple solution (101.43 µM). Hence, the negative control was prepared with 940 μL of DPPH solution and 60 μL of distilled water, and samples consisted of 940 μL of DPPH solution and 60 μL of different concentrations of CBD (31.8, 150, 318, 1590, of 3180 µM) and CBDV (34.91, 174, 349, 1745, or 3491 µM) chosen on the basis of range-finding tests. Furthermore, 940 μL of methanol and 60 μL of distilled water were used for the blank. Trolox was used as the standard control, and methanol was used as the solvent control. The absorbance was measured at 517 nm after 30 min, and the percent decrease in the free radical absorbance was calculated as suggested by Rakholiya et al.^67^:

6 $$\text{Inhibition }\left({\%}\right)=\frac{{\text{OD}}_{517}\text{radical }- {\text{OD}}_{517}\text{sample}}{{\text{OD}}_{517}\text{radical}} \cdot 100$$

The results were expressed as the median effective concentration of sample able to reduce the initial absorbance of the DPPH radical (EC50). Furthermore, TEAC values were calculated in line with Shimamura end colleagues^68^ as previously reported.

### Activity in the presence of bacteria

#### Microbial susceptibility test

*Escherichia coli* (ATCC 13762) and *S. aureus* (ATCC 6538) were cultured overnight in tryptic soy broth and soybean-casein digestive medium (TSB, Oxoid, Milano, Italy) at 37 °C^70^. Subsequently, using 96-well plates (Sarstedt, Italy), 100 µL of TSB, 100 µL of CBD (5–160 µM, DF = 2) or CBDV (5.46–175 µM, DF = 2) and 100 µL of the bacterial culture corresponding to 1 × 10^4^ CFU were added sequentially to each well. CBD and CBDV final concentrations in wells equal to 1.66–53 and 1.82–58.19 µM, respectively, were chosen on the basis of range-finding tests. TSB alone was used as the blank, physiologic solution (0.9% NaCl) was used as the negative control, methanol 5% v/v (final percentage in wells equal to 1.6% v/v) was used as the solvent control, and streptomycin at 60 µM (final concentration in the well equal to 20 µM) was used as the positive control. Experiments were conducted in sextuplicate. The plates were covered with sterile film to prevent evaporation and incubated at 37 °C. After 24, 48 and 72 h, the optical density of each well was recorded at 620 nm using a microplate reader. The bacterial growth inhibition percentage (IC%) was determined as follows:

7 $$\text{IC }\left({\%}\right)=1-\frac{{\text{OD}}_{620}\text{test} \; \text{sample }- {\text{OD}}_{620}\text{blank}}{{\text{OD}}_{620} \text{negative} \; \text{control}-{\text{OD}}_{620}\text{blank }}\cdot 100$$

### Data analysis

All results from five independent experiments are expressed as the IC50 or EC50 as determined by analysis with Prism 5 software (GraphPad Inc., San Diego, CA, USA) and nonlinear regression (log agonist *vs.* normalized response-variable slope) with a 95% confidence range. Lowest observed adverse effect concentrations (LOAECs) were calculated by one-way ANOVA and Dunnett’s comparison test. Differences compared to the controls were considered significant as follows: *p < 0.05, **p < 0.001 and ***p < 0.0001.

## Conclusions

In light of the findings herein, CBD and CBDV are able to cause alterations in the mitochondrial metabolic rate and DNA synthesis in all human-derived cells chosen for this study (both cancer and normal cells) at concentrations on the order of dozens of µmol/L after 72 h of exposure in vitro. A notable difference was observed in membrane damage between cancer and normal cells because the latter, in contrast to the former, were not very sensitive to cannabinoids, probably because of the development of adaptive repair pathways. Furthermore, CBD and CBDV were significantly less active than Trolox in scavenging ABTS and DPPH free radicals. No differences in CBD and CBDV results were observed except for the susceptibility test results, which showed that *S. aureus* was more susceptible to the effects of cannabidiol than to those of cannabidivarin after 72 h of exposure. Thus, cannabinoids can cause DNA damage in human-derived cells, and these molecules can induce general cytotoxicity in both cancer and normal human cells. In addition, the cannabinoids failed to show strong antioxidant capacity. Nevertheless, further in vivo tests may show cell–cell, cell-tissue, tissue-tissue interactions that explain the differing outcomes observed in vitro*.* Moreover, further studies and supplementary assessments are required to determine whether cannabinoids can adequately meet therapeutic expectations without affecting the physiology of the human organism. Additional investigations are imperative for clarifying the molecular mechanisms by which these molecules damage genetic material and would contribute to a better understanding of the potential health risks to humans.

## Supplementary Information

Supplementary Information.
