# Supplementary material for: Comparative assessment of antimicrobial, antiradical and cytotoxic activities of cannabidiol and its propyl analogue cannabidivarin
Source: Sci Rep. 2021 Nov 18;11:22494. doi: 10.1038/s41598-021-01975-z (PMC8602723; doi:10.1038/s41598-021-01975-z)
Supplement: Supplementary file 1 — Supplementary Information. [file 41598_2021_1975_MOESM1_ESM.docx]

Comparative assessment of antimicrobial, antiradical and cytotoxic activities of cannabidiol and its propyl analogue cannabidivarin

Chiara Russo, Margherita Lavorgna, Roberta Nugnes, Elena Orlo and Marina Isidori *

Department of Environmental, Biological and Pharmaceutical Sciences and Technologies, University of Campania “Luigi Vanvitelli”, Via Vivaldi 43, 81100 Caserta, Italy;

[chiara.russo@unicampania.it](mailto:chiara.russo@unicampania.it);

[margherita.lavorgna@unicampania.it](mailto:margherita.lavorgna@unicampania.it);

[roberta.nugnes@unicampania.it](mailto:roberta.nugnes@unicampania.it)

[elena.orlo@unicampania.it](mailto:elena.orlo@unicampania.it)

*Correspondence: [marina.isidori@unicampania.it](mailto:marina.isidori@unicampania.it)

**Table S1.** IC50 (µM) values obtained with MTT assay. IC50 (µM) values obtained with MTT assay, after 24, 48, and 72 h of exposure of different cell lines: A549, Caco-2, Hep G-2, MDA-MB-231 and TelCOFS02MA to CBD and CBDV. Results are expressed means ± standard deviations from five independent experiments. Significant differences (p<0.05) among samples were calculated by One Way-ANOVA, Tukey's Multiple Comparison Test and expressed using different letters **(a, b, c).** Different numbers (from 1 to 5) define different cell lines.

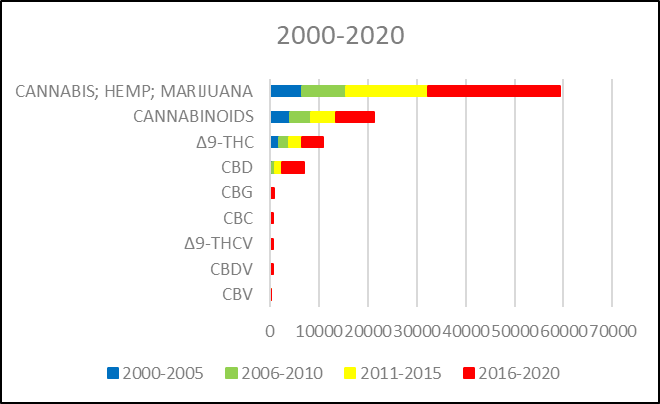


**Figure S1.** Number of scientific documents. Number of scientific documents (https://scifinder.cas.org/) containing “cannabinoids”, ”Δ9-tetrahydrocannabinol“, “cannabidiol”, “cannabigerol“, “cannabichromene“, “Δ9-tetrahydrocannabivarin“, “cannabidivarin”, “cannabivarin” as entered, and at least one of the concepts “cannabis, hemp, marijuana” refined for publication years.


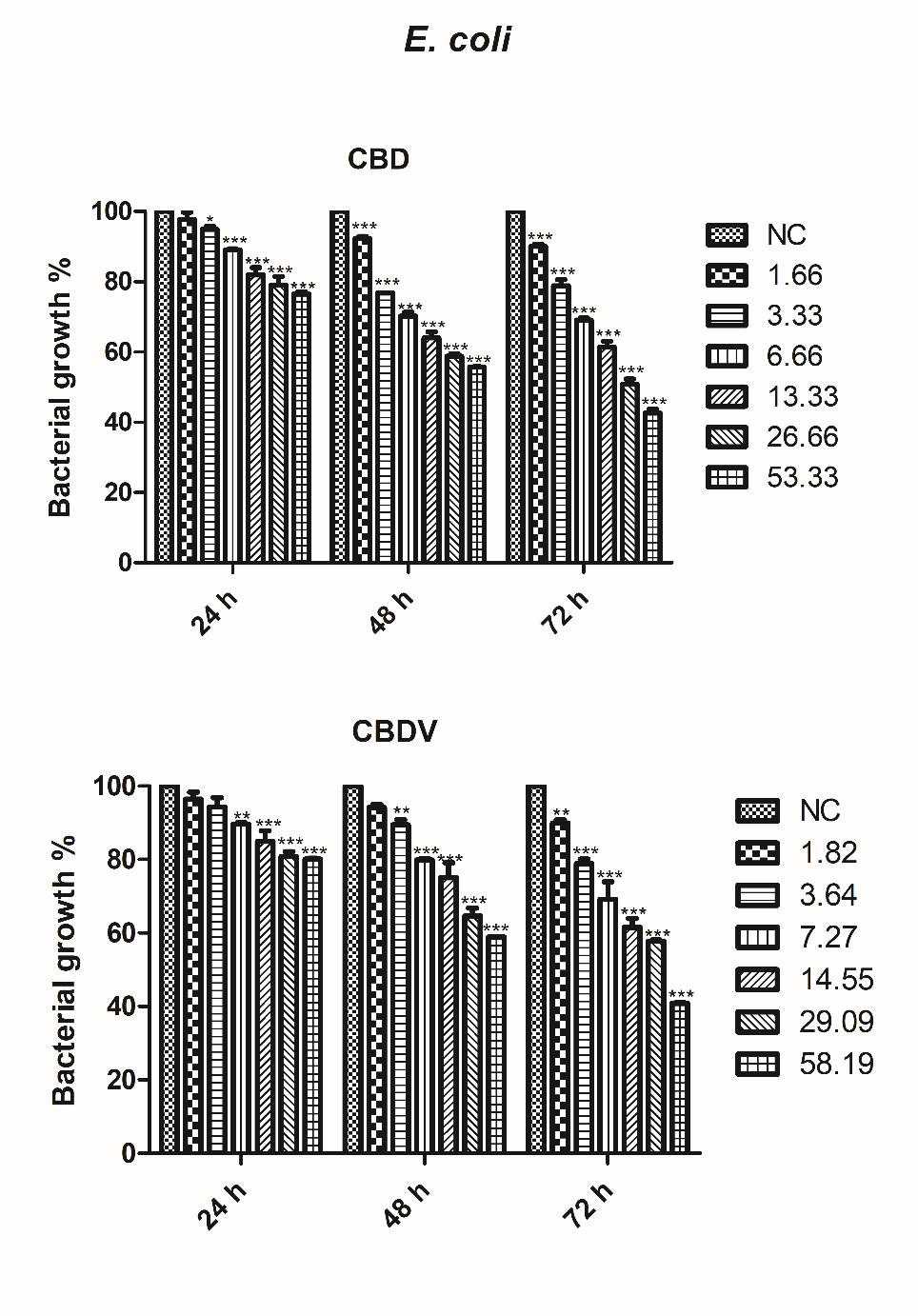


**Figure S2.**

Percentages of *E. coli* growth. Percentages *E. coli* growth after 24-, 48- and 72h- exposure at different concentrations [µM] of CBD or CBDV. Results are expressed as means ± standard deviation from five independent experiments. Significant differences from negative controls are highlighted by asterisks (ANOVA, Dunnett’s test - *p < 0.05; **p < 0.001; ***p < 0.0001).

NC: negative control.

**
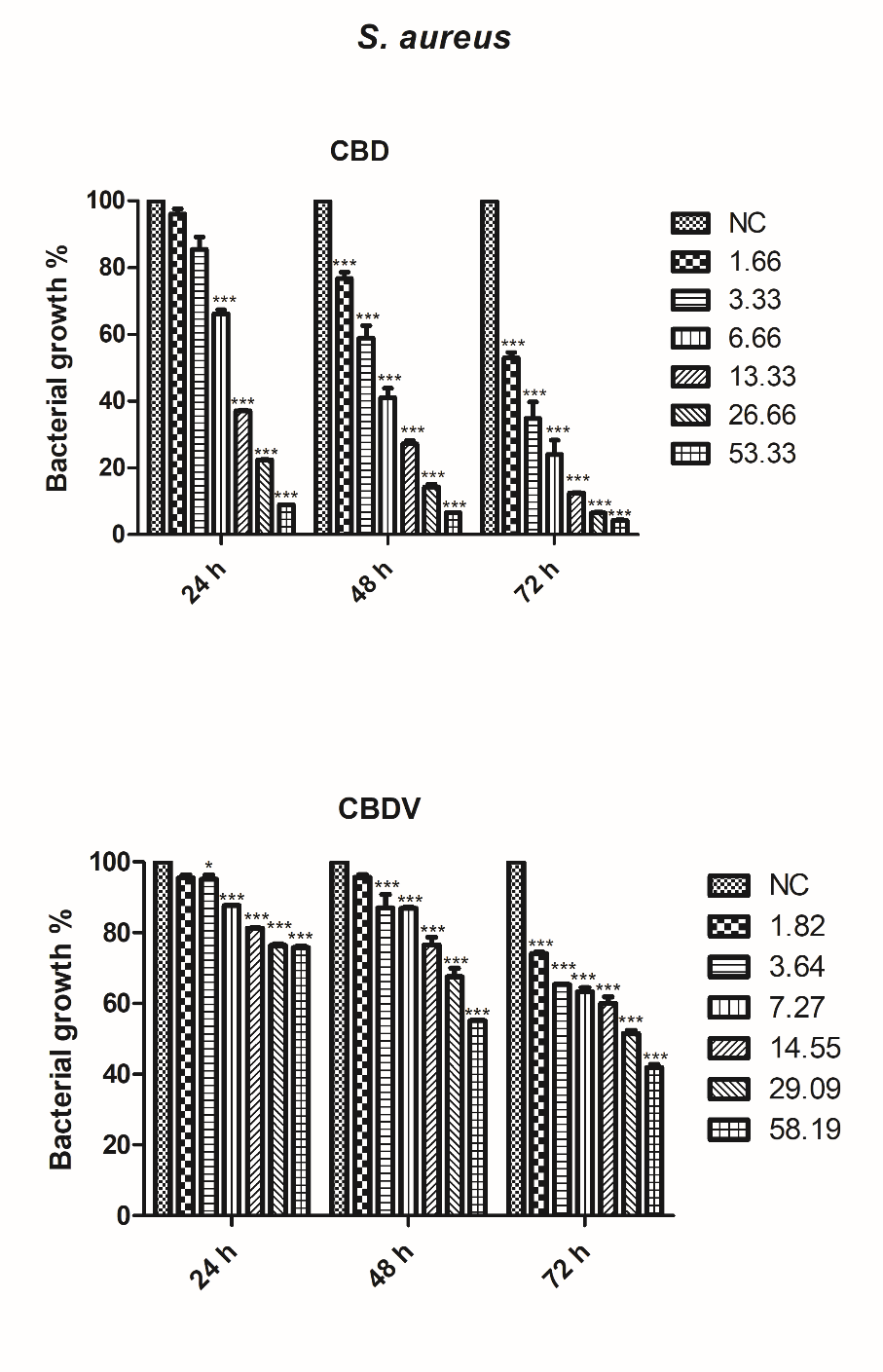
**

**Figure S3.**

Percentages of *S. aureus* growth. Percentages of *S. aureus* growth after 24-, 48- and 72h- exposure at different concentrations [µM] of CBD or CBDV. Results are expressed as means ± standard deviation from five independent experiments. Significant differences from negative controls are highlighted by asterisks (ANOVA, Dunnett’s test - *p < 0.05; **p < 0.001; ***p < 0.0001).

NC: negative control.
